# Immunotherapies and Chemo-Sparing Approaches: A New Era for Diffuse Large B-Cell Lymphoma in Older Patients

**DOI:** 10.3390/cancers18213584

**Published:** 2026-11-07

**Authors:** Filippo Fasano, Bianca Patarnello, Claudia Marinucci, Matteo Bisio, Luca Legato, Barbara Botto, Michele Clerico, Roberto Freilone, Mattia Novo

**Affiliations:** 1 Division of Hematology, A.O.U. Città Della Salute e Della Scienza di Torino, C.so Bramante 88, 10126 Turin, Italy; ffasano@cittadellasalute.to.it (F.F.);; 2 Division of University Hematology, A.O.U. Città Della Salute e Della Scienza di Torino, C.so Bramante 88, 10126 Turin, Italy

**Keywords:** diffuse large B-cell lymphoma, older, immunochemotherapy, monoclonal antibodies, bispecific antibodies, chimeric antigen receptor T-cell therapy

## Abstract

Older patients with diffuse large B-cell lymphoma (DLBCL) represent a particularly challenging population. Reduced-dose rituximab plus cyclophosphamide, doxorubicin, vincristine, and prednisone (R-miniCHOP) can cure approximately half of patients; however, those who fail frontline therapy or are ineligible for anthracycline-based regimens have historically been considered to have a dismal prognosis. Nevertheless, over the past few years, several effective salvage immunotherapies have been introduced into clinical practice, including naked monoclonal antibodies, such as tafasitamab, antibody–drug conjugates, such as polatuzumab vedotin, and loncastuximab tesirine, and CD20 × CD3 bispecific antibodies (BsAbs). Moreover, novel combined approaches are currently under investigation as frontline therapy, such as the triplets polatuzumab vedotin plus rituximab and glofitamab (Pola-R-Glo), and tafasitamab plus lenalidomide and rituximab (TLR), or the upfront use of single-agent CD20 × CD3 BsAbs. These strategies aim to spare toxicity in older and frail patients and have shown promising efficacy, potentially heralding a chemo-free era for DLBCL in the older in the near future.

## Simple Summary

The treatment of diffuse large B-cell lymphoma in older patients can be particularly challenging; although a reduced-intensity immunochemotherapy regimen is the actual standard of care, safer and more effective approaches are needed, especially for frail patients and for those who fail initial treatment. In this review, we examine the growing number of chemotherapy-free and chemotherapy-sparing strategies being developed for older patients, such as targeted antibodies, antibody–drug conjugates, immune-modulating drugs, bispecific antibodies, and cellular therapies, either alone or in combination. We discuss their efficacy, safety, and potential application, with the aim of clarifying how they may be integrated into clinical practice. These innovative approaches may provide effective disease control while reducing treatment-related toxicity, and may contribute to a future paradigm in which conventional chemotherapy can be substantially reduced or avoided.

## 1. Introduction

Diffuse large B-cell Lymphoma (DLBCL) is potentially curable with standard frontline immunochemotherapy with rituximab, cyclophosphamide, doxorubicin, vincristine and prednisone (R-CHOP) achieving long-term disease control in approximately 60% of cases [1,2].

However, outcomes remain less favourable in the relapsed or refractory (R/R) setting, where treatment strategies have traditionally relied on salvage chemotherapy followed by autologous stem cell transplantation (ASCT) in eligible patients, particularly younger individuals [3,4].

In the past few years, the therapeutic landscape for R/R DLBCL has improved substantially, largely owing to the introduction of CD19-directed chimeric antigen receptor T-cell (CAR-T) therapy [5,6,7]. More recently, polatuzumab vedotin in combination with rituximab, cyclophosphamide, doxorubicin and prednisone (Pola-R-CHP) has been approved for untreated DLBCL patients thanks to the positive results of the phase 3 POLARIX trial (NCT03274492; median age 65 years, range 19–80), which demonstrated a significant improvement in progression-free survival (PFS) compared with R-CHOP, but no statistically significant benefit in overall survival (OS); PFS benefit was also observed in patients aged ≥60 years, supporting the potential role of the treatment regimen in this population [8]. A promising perspective for future frontline treatment strategies may also emerge from the more recent phase 3 frontMIND trial, in which the addition of tafasitamab and lenalidomide to R-CHOP improved PFS in patients with high-risk DLBCL [9].

Until recently, older patients with DLBCL have had poorer outcomes: reduced-intensity CHOP combined with rituximab (R-miniCHOP) has been considered the standard of care (SOC) for patients aged >80 years, achieving cure rates of approximately 50% [10,11].

Durable treatment responses have remained difficult to achieve in patients with R/R disease, as advanced age and comorbidities often limit eligibility for intensive therapeutic strategies. However, emerging evidence suggests that CAR-T cell therapy may represent a safe and feasible option for carefully selected patients [12]. In addition, for very old patients considered unsuitable for anthracycline-based therapy, the treatment approach is considered palliative from the outset. Nevertheless, over the last decade several novel agents and combination regimens have been approved for older patients, including antibody–drug conjugates (ADCs), naked monoclonal antibodies (mAbs), and CD20 × CD3 bispecific antibodies (BsAbs), reshaping the outcomes in this subset of patients [13].

In this review, a literature search was conducted to identify relevant studies investigating emerging chemotherapy-sparing and chemotherapy-free therapeutic strategies for older patients with DLBCL, in both the frontline and R/R settings. Electronic databases, such as PubMed, Embase, and Scopus, were searched for articles published up to August 2026 using a combination of MeSH terms and keywords, including “diffuse large B-Cell lymphoma,” “frontline,” “relapsed,” “refractory,” “treatment,” “older,” “elderly,” and “immunochemotherapy.” The reference lists of relevant retrieved articles were also manually screened to identify additional studies of interest (secondary searching). In parallel, the official websites of the European and US regulatory agencies were consulted to retrieve relevant information on the approval status of selected therapeutic agents. ClinicalTrials.gov was systematically searched to identify ongoing clinical trials investigating chemotherapy-containing, chemotherapy-sparing, or chemotherapy-free approaches in the same patient population, including them in a dedicated table. Original graphical materials were created using BioRender and R software (version 4.6.1).

### Geriatric Assessment

Treatment selection in older patients with DLBCL remains challenging, as chronological age alone is an insufficient surrogate for physiological reserve, cognitive and nutritional status, functional independence, comorbidity and polypharmacy burden, and treatment tolerance; a comprehensive assessment of individual fitness becomes essential to identify patients who may benefit from full-dose immunochemotherapy, and those for whom less intensive or chemotherapy-free approaches may be more appropriate. In this setting, the simplified geriatric assessment (sGA) developed within the Fondazione Italiana Linfomi (FIL) Elderly Project represents a practical tool for guiding clinical decision-making. In this prospective study of 1163 patients aged ≥65 years, the sGA integrated age, comorbidity burden, and functional autonomy, assessed through the Cumulative Illness Rating Scale for Geriatrics (CIRS-G), activities of daily living (ADL), and instrumental activities of daily living (IADL). Patients were subsequently classified as fit, unfit, or frail, accounting for 55%, 28%, and 18% of the study population; this stratification identified groups with markedly different outcomes, with 3-year OS rates of 75%, 58%, and 43%, respectively, supporting the prognostic relevance of a multidimensional assessment beyond chronological age alone [14].

## 2. Current and Upcoming Options for R/R Patients

### 2.1. Second-Line Options

#### 2.1.1. Polatuzumab Vedotin Plus Bendamustine and Rituximab (Pola-BR)

Polatuzumab vedotin is an ADC targeting CD79b, a key component of B-cell receptor signalling, and conjugated to monomethyl auristatin E (MMAE), a microtubule inhibitor. A study by L.H. Sehn et al. [15] supported its accelerated approval in combination with bendamustine and rituximab (Pola-BR) for non-transplant eligible (NTE) patients with R/R DLBCL. The regimen was first approved by U.S. Food and Drug Administration (FDA) in 2019, followed by the European Medicines Agency (EMA) and other regulatory authorities. In this phase 2 randomized trial (NCT02257567) the combination Pola-BR (median age 67 years, range 33–86) demonstrated superior overall response rate (ORR), complete response (CR), PFS, and OS compared with BR alone (median age 71 years, range 30–84), while maintaining an acceptable and manageable safety profile [15]. These results were further corroborated by an extension cohort comprising more than one hundred patients (median age 70 years, range 24–94), with a median follow-up of 15.2 months. The study reported an ORR of 41.5%, median PFS and OS of 6.6 months and 12.5 months, respectively, and median duration of response (DOR) of 9.5 months. The clinical benefit of polatuzumab vedotin appeared to be preserved regardless of the number of previous treatment lines or refractory status to the previous treatment. Nevertheless, a trend towards more favourable outcomes was observed in patients who received Pola-BR earlier in the treatment course and in those who were not refractory to their previous treatment [16].

Several real-world studies, characterized by heterogeneous patient populations and treatment settings, further explored the role of Pola-BR in routine clinical practice. The combination confirmed to be well tolerated, with neuropathy as main treatment-emergent adverse event (AE). ORR ranged from 48% to 73% with CR rates of 40–45%, while survival data were partially discordant among studies, with median PFS ranging from 3 to 14 months and median OS of 5 to more than 12 months, likely reflecting the intrinsic patients and treatment variability in routine clinical practice. In general, multivariate analyses identified bulky disease, more than one prior line of therapy, elevated Eastern Cooperative Oncology Group (ECOG) performance status, elevated lactate dehydrogenase (LDH) levels, and refractoriness to previous treatments as the main factors associated with inferior outcomes [17,18,19,20,21,22,23,24].

#### 2.1.2. Tafasitamab Plus Lenalidomide (Tafa-Len)

The combination tafasitamab plus lenalidomide represents a chemo-free option recently introduced in clinical practice for NTE R/R DLBCL, available from the second line. Tafasitamab is a naked mAb targeting CD19, a transmembrane protein playing a central role in B-cell biology, inducing tumor cell lysis both directly, and through recruitment of immune effector cells. Its synergistic activity with lenalidomide, a pleiotropic immunomodulatory agent, was evaluated in 80 patients enrolled in the phase 2 L-MIND trial (NCT02399085; median age 72 years, range 41–86). Tafa-Len demonstrated an ORR of 57.5% (CR, 41.3%) with a median time to response of 2 months, and durable responses especially for patients achieving CR: at the 5-year analysis median PFS and OS were of 11.6 and almost 34 months, respectively. At 44 months of follow-up, the median DOR had not been reached, with an estimated 5-year duration of CR of 80.7%. The treatment was well tolerated overall, with most commonly reported AEs including cytopenias (particularly neutropenia), diarrhea, rash, and infections, whose incidence tended to decrease following transition to tafasitamab monotherapy [25,26].

Although the first real-life data reported following Tafa-Len approval were relatively disappointing, other subsequent real-word evidence studies (RWE) showed efficacy more consistent with that observed in the pivotal trial. The first RWE study presented, including 178 heavily pretreated patients (median age 75 years, range 26–94), most of whom were ineligible according to the L-MIND trial criteria, reported a best ORR of 31%, with CR achieved in 19% of patients, with a median PFS and OS of 1.9 and 6.5 months, respectively [27].

In a second large retrospective multicenter US cohort including 181 patients (median age 71 years, IQR 65–76), the ORR was 73% (CR, 23%; PR, 50%), with a median DOR of 9.6 months, and 19.2 months for CRs; median PFS and OS were 11.3 and 24.8 months, respectively. Improved outcomes were observed among patients receiving Tafa-Len as second-line therapy rather than in later treatment lines [28].

Among 83 patients (median age 78 years, range 47–93) treated with Tafa-Len in a Spanish cohort, and followed for a median of 21.6 months, ORR was 61% (CR, 42%), with a not-reached median DOR, and a median OS of 21.8 months. Maintaining an adequate lenalidomide relative dose intensity, at least 70%, was statistically associated with improved DOR and PFS, supporting the importance of optimal supportive care measures in routine clinical practice [29].

The multicenter retrospective EarlyMIND French study on 186 patients (median age 78 years, range 32–93) reported greater efficacy when administered as second-line therapy, achieving a best ORR of 46.8% (CR, 29%), median PFS and OS of 4.7 and 10 months, respectively, and confirming durable responses among patients achieving CR [30].

Additional real-world experiences have reported broadly comparable findings, generally confirming that Tafa-Len efficacy remains related to patients and disease characteristics [31,32].

### 2.2. Treatment Options as Third or Subsequent Lines

#### 2.2.1. Loncastuximab Tesirine

Loncastuximab tesirine (lonca) is an ADC comprising a humanized anti-CD19 mAb conjugated to a pyrrolobenzodiazepine dimer toxin through a cathepsin-cleavable linker [33].

The multicenter phase 2 LOTIS-2 trial (NCT03589469) evaluated the safety and antitumour activity of lonca in 145 patients (median age 66 years, range 23–94) with R/R DLBCL who had received two or more prior lines of systemic therapy. The study enrolled a heavily pretreated population, including 9% of patients previously exposed to CAR-T cells. A substantial proportion of participants also presented with high-risk disease characteristics, including double- or triple-hit lymphoma, transformed DLBCL, or primary refractory disease. Lonca demonstrated an ORR of 48.3%, with CR of 24.1%. The median DOR was 10.3 months, increasing to 13.4 months among patients achieving CR. Notably, 64% of responders maintained their response at 9 months. The median PFS and OS were 4.9 and 9.9 months, respectively. The safety profile was manageable: the most common grade (G) ≥ 3 AEs being neutropenia (26%), thrombocytopenia (18%), and elevated gamma-glutamyltransferase (17%). Toxicities associated with the pyrrolobenzodiazepine payload, including oedema, skin reactions, and liver enzyme elevations, were generally mild to moderate in severity and reversible [34]. These encouraging findings supported the FDA and EMA approval of lonca for adult patients with R/R DLBCL and high-grade B-cell lymphoma (HGBL) following two or more prior lines of systemic therapy [35,36].

The favourable safety profile of lonca has been demonstrated by its comparison with the regimen Pola-BR in a matching-adjusted indirect comparison (MAIC) study, which evaluated the relative efficacy and safety of the two treatments in patients with R/R DLBCL. While no statistically significant differences were observed in key efficacy endpoints, lonca showed significantly lower odds of grade ≥3 infections and other AEs compared to Pola-BR, supporting its suitability for older and frailer patients [37].

#### 2.2.2. Bispecific Antibodies

Bispecific antibodies (BsAbs) are a diverse family of engineered molecules composed of two linked antibody fragments that can bind two distinct epitopes simultaneously. Most BsAbs currently in clinical use for B-cell neoplasms belong to the class of Bispecific T-cell Engagers (TCEs), which simultaneously bind the CD3 antigen on the T lymphocyte and a tumor-associated antigen, such as CD19 or CD20, inducing cytotoxic synapse formation and target-cell killing, independently of T-cell receptor (TCR) specificity. Three CD20 × CD3 BsAbs products demonstrated consistent efficacy and good tolerability for treatment of multirefractory DLBCL, and they are currently available in clinical practice with no upper age limit in their approved indications; while glofitamab and epcoritamab are approved by both US and European regulatory authorities, odronextamab is currently approved by the EMA but not the FDA.

##### Glofitamab

Glofitamab is a full-length BsAb constructed on an IgG1 Fc (fragment crystallisable) backbone and features a novel 2:1 fragment antigen binding (Fab), incorporating two CD20 binding domains and one CD3 binding domain, potentially conferring a superior antitumor activity.

The efficacy and safety of glofitamab monotherapy were evaluated in a phase 2 study (NCT03075696) involving patients with R/R DLBCL who had undergone at least two previous lines of therapy. The study enrolled 155 patients with a median age of 66 years (range 21–90) and a median of three prior lines of therapy; notably, 33% had previously received CAR-T treatment. Patients received obinutuzumab pretreatment to mitigate the risk of cytokine release syndrome (CRS), followed by fixed duration glofitamab monotherapy for a total of 12 cycles. The treatment demonstrated substantial clinical activity yielding an ORR of 52% and a CR rate of 39%. Efficacy was also observed in the subgroup of patients previously treated with CAR-T, in whom the CR rate reached 35%. The median DOR was 18.4 months, while 78% of patients achieving CR maintained their remission at 12 months [38]. Regarding safety, CRS was reported in 63% of patients, although severe events (grade ≥ 3) accounted for fewer than 5% of events. Immune effector cell-associated neurotoxicity syndrome (ICANS) occurred in 7.8% of patients, with grade ≥ 3 events observed in 2.6%. Overall, these findings indicated a manageable safety profile, supporting the clinical applicability of glofitamab, including in a older patient population. On the basis of these results, glofitamab received FDA and EMA approval for the treatment of R/R DLBCL following at least two previous lines of therapy [39,40].

The 3 years follow-up confirmed the durability of responses achieved with fixed-duration treatment: the median DOCR reached 29.8 months, and all patients with ongoing remission had been treatment-free for at least 2 years following end of treatment (EOT). In patients with a CR at EOT, the PFS and OS rates 2 years after EOT were 57% and 77%, respectively. These results are particularly interesting considering older patients who are generally not eligible for consolidation therapies. The long-term safety profile remained favourable [41].

##### Epcoritamab

Epcoritamab is a subcutaneously administrated IgG1 based CD20 × CD3 BsAb, approved by both the FDA and EMA in 2023 for the treatment of R/R DLBCL after two or more lines of therapy [42,43].

The dose escalation phase of the multicenter, phase 1–2 EPCORE NHL-1 trial (NCT03625037) established the promising single-agent antitumour activity and favourable safety profile of epcoritamab in patients with B-cell non-Hodgkin lymphomas (B-NHLs) treated until disease progression [44]. In the dose-expansion cohort, which included 157 patients (median age 64 years, range 20–83) with R/R large B-cell lymphoma (LBCL) previously exposed to at least two prior lines of therapy, epcoritamab achieved an ORR of 63.1% and a CR rate of 38.9% at a median follow-up of 10.7 months. Interestingly, efficacy was maintained across all age groups, with ORR of 56.3%, 68.8%, and 72.4% and CR rates of 35.0%, 39.6%, and 48.3% in patients aged <65, 65–75, and ≥75 years, respectively. CRS occurred in 49.7% of patients and was predominantly grade 1–2 (47.1%), whereas grade 3 events were uncommon (2.5%) and no grade ≥ 4 events were reported. ICANS was rare, occurring in 6.4% of patients, with most events classified as grade 1–2 [45]. Updated results at the 3-year follow-up confirmed the durability of responses, with a CR rate of 41%, a median DOCR of 36.1 months, and 52% of CR still ongoing. The median OS was 18.5 months in the overall population, while it was not reached in complete responders [46]. Recently, a post-hoc analysis demonstrated that patients with R/R LBCL in CR can maintain durable remissions even after temporary treatment pauses or permanent discontinuation of epcoritamab, suggesting a degree of flexibility in the epcoritamab treatment regimen for patients achieving deep responses [47].

##### Odronextamab

Odronextamab is a IgG4-based CD20 × CD3 BsAb engineered with specific Fc-region mutations, which in 2024 received EMA approval for the treatment of R/R DLBCL after two or more lines of systemic therapy [48].

The phase 1, single-arm, multicentre ELM-1 trial (NCT02290951) evaluated the antitumour activity of intravenous odronextamab in 145 heavily pretreated patients (median age 67 years, IQR 57–73) with R/R B-NHL, including 85 patients with DLBCL. Odronextamab was administered using a step-up dosing schedule in cycle 1, followed by an until-progression-treatment schedule. In patients with DLBCL receiving doses of ≥80 mg, the ORR was 53% (all CR) and 33% (27% CR) for CAR-T naïve and CAR-T exposed patients, respectively. The safety profile was generally manageable, with CRS occurring in 61% of patients but predominantly low grade (G 1–2: 54%; G ≥ 3: 7%) [49].

The ongoing, multicohort, phase 2 ELM-2 study (NCT03888105) found further odronextamab efficacy in 127 R/R DLBCL (median age 67 years, range 24–88), with a longer follow-up: 52% ORR (50% in patients > 75 years, and 46% in those with more than two lines of prior therapy), 31% CR, and a median DOCR of 17.9 months [50].

### 2.3. Novel Second-Line Options

#### 2.3.1. Glofitamab Plus Gemcitabine and Oxaliplatin (Glofi-GemOx)

The efficacy of glofitamab combined with gemcitabine plus oxaliplatin (Glofi-GemOx) was assessed in the large phase 3 STARGLO trial (NCT04408638). The 274 enrolled patients with R/R DLBCL (median age 68 years, IQR 58–74), had received at least one prior line of therapy and were NTE. They were randomized 2:1 to receive Glofi-GemOx (*n* = 183) or a SOC approach consisting of rituximab plus gemcitabine and oxaliplatin (R-GemOx) (*n* = 91). Regardless of the high proportion of heavily pretreated patients, the experimental cohort demonstrated clear improvements in median OS (25.5 vs. 12.9 months, primary endpoint), median PFS (13.8 vs. 3.6 months), ORR (68.3% vs. 40.7%) median DOR (not reached vs. 10.3 months), as well as CR rate (58.5% vs. 25.3%) and CR duration (not reached vs. 24.2 months), despite a more complex safety profile including serious infections in 26% of the patients (vs. 13%). Transient CRS and ICANS occurred in 44% and 2% of patients treated with glofitamab, respectively, predominantly as low-grade events [51]. In light of these positive results, Glofi-GemOx has been approved by EMA in 2025 as treatment option for NTE R/R DLBCL, thus anticipating the availability of a BsAb in the second line [40]. By contrast, the FDA did not approve the regimen, citing concerns about the generalizability of the STARGLO findings to the US population given the limited number of US patients enrolled, their uneven geographic distribution, and the lack of representativeness of the US population [52].

The 3-year follow-up endorsed the sustained superiority of Glofi-GemOx over SOC, with a pronounced efficacy in the second-line setting and across age groups, including patients aged ≥75 years; no new safety signals emerged, and the overall toxicity profile remained manageable and consistent with previous findings [53].

#### 2.3.2. Mosunetuzumab Plus Polatuzumab Vedotin (Mosun-Pola)

Mosunetuzumab, another CD20 × CD3 BsAb, has demonstrated single-agent activity with an ORR of 42.0% and a CR rate of 23.9% in 88 heavily pretreated R/R DLBCL (NCT02500407) [54]. Building on these findings, mosunetuzumab was subsequently investigated in combination with polatuzumab vedotin in a phase 1b/2 study (NCT03671018); among 98 patients in the dose-expansion cohort (median age 68 years, range 20–88), the combination achieved an ORR of 59.2% and a CR rate of 45.9%, with a median PFS of 11.4 months after a median FU of 23.9 months [55].

Subsequently, a randomized phase 2 cohort within the same clinical program compared Mosun-Pola (median age 72 years, range 36–87) with R-Pola (median age 68 years, range 24–92) in 80 patients randomized 1:1. Mosun-Pola achieved higher ORR (77.5% vs. 50.0%) and CR rates (55.0% vs. 35.0%) than R-Pola, with median PFS of 25.4 versus 6.4 months, respectively [56].

These findings led to further evaluation in the phase 3 SUNMO trial (NCT05171647), which randomized 208 patients (median age 62 years, range 23–87) with NTE R/R LBCL in a 2:1 ratio to receive Mosun-Pola or R-GemOx. After a median FU of 23.2 months, the experimental arm showed significantly improved ORR (70% vs. 40%) and CR rate (51% vs. 24%), as well as median PFS (11.5 vs. 3.8 months). The combination had a manageable safety profile, with an overall incidence of AE similar between groups. Neutropenia was observed in roughly one third of patients as the most common grade ≥ 3 AE, whereas severe thrombocytopenia and anemia were less frequent with Mosun-Pola than with R-GemOx (2.2% vs. 36% and 5.9% vs. 19%, respectively), as well as peripheral neuropathy (24% vs. 42%); on the other hand, infections of any grade were more frequent (51% vs. 31%) in the Mosun-Pola arm. Grade ≥ 2 CRS occurred in fewer than 5% of patients, and no ICANS events were observed [57].

Mosun-Pola may represent a novel second-line option, although it has not yet received approval from US or European regulatory authorities.

#### 2.3.3. Brentuximab Vedotin Plus Lenalidomide and Rituximab (BV-Len-R)

The phase 3 ECHELON-3 trial (NCT04404283) investigated brentuximab vedotin, an anti-CD30 ADC, in combination with lenalidomide and rituximab in patients with NTE R/R DLBCL who received at least two previous lines of therapy. 230 patients were randomized to BV-Len-R (*n* = 112; median age 74 years, range 29–87) or placebo plus lenalidomide and rituximab (*n* = 118; median age 70 years, range 21–89). At a median follow-up of 16.4 months, BV-Len-R significantly improved median OS (13.8 vs. 8.5 months) and PFS (4.2 vs. 2.6 months). The ORR was 64% versus 42%, with CR rates of 40% and 19%, respectively, corresponding to a 47% reduction in the risk of disease progression or death with the experimental regimen. Interestingly, the OS benefit was maintained across most subgroups, including patients aged ≥65 years. However, this benefit came at the expense of greater toxicities: in the BV-Len-R arm grade ≥ 3 treatment-emergent AEs were documented in 88% of the cohort (vs. 77%), with the most common being hematological ones and diarrhea. Serious AEs were more frequent in the experimental arm too (60% vs. 50%), mainly related to infections; peripheral neuropathy was mainly low-grade and overall observed in 31% of the patients (vs. 24%) [58].

Overall, BV-Len-R represents a further option for heavily pretreated patients with R/R DLBCL, although its regulatory approval for this indication is currently limited to the US.

### 2.4. CAR-T Cells

#### 2.4.1. Role in Transplant Ineligible/CAR-T Eligible Patients

The recent advent of CAR-T cell therapies has significantly expanded the treatment options available for patients with high-risk R/R LBCL. Axicabtagene ciloleucel (axi-cel), lisocabtagene maraleucel (liso-cel), and tisagenlecleucel (tisa-cel) have shown benefit and manageable safety profiles in their pivotal trials which also included patients aged ≥75 years. Therefore, they may be considered a treatment option for selected patients who are considered NTE based on the assessment of physiological fitness, performance status, comorbidities, organ function, and the ability to tolerate high-dose chemotherapy and the transplant procedure.

The ZUMA-1 study (NCT02348216), which evaluated axi-cel as third-line or later therapy in 101 patients with R/R LBCL (median age 58 years, range 23–76), reported the long-term results after a median follow-up of 27 months: ORR was 83% (CR, 58%) and the estimated 2-year PFS rates were 72% and 75%, among patients with CR and PR at 3 months, respectively. Of note, 24 patients were aged ≥65 years and demonstrated a median PFS comparable to that of patients aged <65 years [5]. TRANSCEND NHL 001 (NCT02631044) is the pivotal phase 2 study evaluating liso-cel as third or subsequent line for 269 patients with R/R LBCL. The median age of the population was 63 years (range 18–86), with 112 patients ≥ 65 years, and 27 patients ≥ 75 years. Data from the extended follow-up indicated that liso-cel maintained durable clinical activity with 2-year PFS and OS rates of 40.6% and 50.5%, respectively, in a broad population of patients. The ORR was 73%, with 53% of patients achieving CR. Moreover, liso-cel infusion was associated with low incidences of severe CRS or ICANS [6,59].

The phase 2 JULIET trial (NCT02445248) evaluated tisa-cel in patients with R/R LBCL after multiple prior lines of therapy. Among the 115 infused patients (median age, 56 years), 23% were aged ≥65 years. The ORR was 53%, with a CR rate of 39%. At a median follow-up of 74.3 months in the extended analysis, the estimated 5-year PFS and OS rates were 28% and 32%, respectively [7,60].

Subsequently, ZUMA-7 (NCT03391466) and TRANSFORM (NCT03575351) phase 3 trials, established greater efficacy of axi-cel and liso-cel as second-line therapy compared with the SOC, consisting of platinum-based immunochemotherapy followed by ASCT. In addition, they demonstrated improved benefit with respect to their use in third-line or later, still maintaining a similar safety profile. Of note, the median age of the patients enrolled in the two studies was 58 years and 60 years in ZUMA-7 and TRANSFORM, respectively. However, the age ranges were 21–80 years and 20–74 years, respectively, with more than 30% of patients aged >65 years [61,62,63].

#### 2.4.2. Future Perspective for Older Patients

Although the pivotal above-mentioned studies were not specifically designed with the older population as the primary target, both therapies showed improved efficacy and tolerability compared with standard treatment in this subgroup as well. In this context, the ALYCANTE and PILOT trials were conducted to evaluate the efficacy and safety of CAR-T therapy as a second-line treatment in patients with high-risk NTE R/R LBCL.

Phase 2 ALYCANTE trial (NCT04531046) treated 62 patients with R/R LBCL (median age 70 years, range 49–81) and considered ineligible for ASCT, with axi-cel in second-line. Almost all patients had an ECOG performance status of 0 or 1. The primary end point of the study was met with a CR of 71% at 3 months. With a median follow-up of 12 months, median PFS was 11.8 months and OS was not reached. There was no unexpected toxicity; grade ≥ 3 CRS and ICANS occurred in 8.1% and 14.5% of patients, respectively. The efficacy of axi-cel was maintained across key subgroups, including patients with high-risk features, such as age ≥70 years, and primary refractory disease [64].

In the PILOT trial (NCT03483103) 61 adults with NTE R/R LBCL who had received one previous line of therapy were treated with liso-cel. The median age of the population was 74 years (range 53–84). The ORR was 80%, and 54% of patients achieved CR. After median follow-up of 18.2 months, median DOR was 23.3 months. The 18-month PFS and OS rates were 43% and 59%, respectively. In the treatment emergent (TE) period (≤90 days after liso-cel administration), 79% had grade ≥ 3 AEs with 38% of CRS (G3: 2%; no G4–5), and 7% had grade ≥ 3 infections. In the post-TE period, 18% of patients experienced grade ≥ 3 AEs with only one patient experiencing grade ≥ 3 infection. In this population with high incidence of primary refractory disease, advanced age, and comorbidities, liso-cel demonstrated durable efficacy and a favourable safety profile, consistent with previous reports [65].

All together these studies continue to support liso-cel and axi-cel as second-line options for this underserved population and have paved the way for broader access to CAR-T therapy in older patients. As access expands, eligibility should be assessed on a multidisciplinary and personalized approach that considers the disease setting and product-specific indication, performance status, organ function, comorbidity burden, functional and cognitive status, and toxicity management, as chronological age alone should not be considered an absolute criterion.

More recently, zamtocabtagene autoleucel (zamto-cel) has emerged as a promising novel CAR-T option for patients who are ineligible for ASCT. Zamto-cel is a non-cryopreserved, tandem CD19/CD20-targeted CAR-T cell product manufactured using a rapid, fully automated process. It was evaluated in the phase 2 DALY 2-EU (NCT04844866) randomized trial versus immunochemotherapy (R-GemOx or Pola-BR) as second-line treatment for NTE patients with relapsed or early relapsed (≤24 months) LBCL. In the primary analysis, conducted in a vulnerable older population with a median age of 74 years (range 19–87), zamto-cel demonstrated clinically meaningful superiority over immunochemotherapy, achieving an ORR of 72% and a CR rate of 54% (vs. ORR 45% with CR 15% for the standard arm), and a significantly longer median PFS of 8.5 months (vs. 3.3 months), while maintaining low rates of severe toxicities (CRS G ≥ 3: 5.3%; ICANS G ≥ 3: 1.3%) [66].

### 2.5. Novel Combinations Under Development

#### 2.5.1. Epcoritamab Plus Lenalidomide (E-Len)

Among the various combination strategies being explored for monoclonal antibody-based regimens, epcoritamab plus lenalidomide represents a promising chemo-free approach, with early data suggesting encouraging antitumor activity and a manageable safety profile.

In the Arm 1 of ongoing phase 1b/2, multi-arm, EPCORE NHL-5 trial (NCT05283720), patients with NTE R/R DLBCL, or who had failed it, received fixed-duration epcoritamab plus lenalidomide (E-Len) for up to twelve 28-day cycles. Updated results, based on 40 treated patients (median age 71.5 years, range 26–85) with a median follow-up of 11.5 months, showed an ORR of 67.6% and a CR rate of 51.4%, with median DOR not yet reached, and a manageable, predictable safety profile, with a CRS rate of 65%, primarily low grade [67]. These encouraging results supported the design of the ongoing phase 3 EPCORE DLBCL-4 trial (NCT06508658), which is evaluating the E-Len as second-line or later therapy in a randomized setting against R-GemOx or epcoritamab single-agent.

#### 2.5.2. Loncastuximab Tesirine in Combined Modalities

The LOTIS-5 trial (NCT04384484)—an ongoing phase 3, randomized, open-label, multicenter study—is evaluating the efficacy of the combination lonca plus rituximab (Lonca-R) compared to standard immunochemotherapy (R-GemOx) in NTE patients with R/R DLBCL after ≥1 prior line of therapy. Approximately 330 patients have been randomized 1:1 to receive Lonca-R or R-GemOx for up to 8 cycles [68]. Enrollment was completed in December 2024, and topline results of the primary endpoint analysis are pending. In the non-randomized safety run-in, which included 20 LBCL patients (median age 74.5 years, range 35–93), Lonca-R demonstrated promising antitumor activity with an ORR of 80% and a CR rate of 50%, a median PFS of 8.3 months, and a median DOR of 8.0 months, with CR duration not reached. The safety profile was manageable, with grade ≥ 3 AEs occurring in 11 patients (most commonly increased GGT and neutropenia) [69].

The efficacy and safety profiling of lonca plus glofitamab (Lonca-Glofi) in patients with R/R LBCL is currently being investigated by the ongoing, phase 1b, LOTIS-7 trial (NCT04970901). The 31 patients evaluated, with a median age of 73 years and the majority having received ≥2 prior lines of therapy, showed an ORR of 95.5% (CR, 90.9%), with the median DOR not reached at the time of analysis. The combination showed a manageable safety profile, with no grade ≥ 3 CRS or ICANS. Pharmacodynamic analyses demonstrated T-cell margination and activation, supporting the potential complementary activity of the two agents [70].

## 3. Promising Low-Intensity Approaches in First-Line Therapy

In recent years, several intriguing trials have been designed with the aim of substantially reducing or eliminating the chemotherapy backbone from first-line treatment for older patients with DLBCL.

### 3.1. Polatuzumab Vedotin Plus Rituximab and miniCHP (Pola-R-miniCHP)

Pola-R-CHP regimen recently demonstrated superior efficacy compared with R-CHOP in the phase 3 POLARIX trial and has become the new SOC in many countries as first-line treatment for younger patients (≤80 years) with intermediate- to high-risk DLBCL (IPI ≥ 2) [8].

To date, several retrospective studies have reported encouraging efficacy and an acceptable safety profile for attenuated-dose pola-R-CHP in older patients [71,72,73,74].

The ongoing phase 3 POLAR BEAR trial (NCT04332822) is randomizing older or frail patients to receive either a dose-reduced Pola-R-CHP (Pola-R-miniCHP) or R-miniCHOP. With a median follow-up of 11.4 months, the initial safety analysis of 127 patients demonstrated a comparable safety and tolerability profile between the two treatment arms, except for a higher incidence of predominantly low-grade gastrointestinal AEs in the experimental arm. The final results of the study are pending and may support the adoption of pola-R-miniCHP as frontline treatment in older patients in the near future [75].

### 3.2. Polatuzumab Vedotin Plus Rituximab and Glofitamab (Pola-R-Glo)

The combination of polatuzumab vedotin, rituximab, and glofitamab has been investigated in a phase 2 trial (NCT05798156) involving previously untreated frail patients aged ≥60 years with DLBCL who were considered unsuitable for full-dose R-CHOP.

The primary analysis of the study has recently been presented. Among 80 patients with a median age of 80 years (range 66–92), EOT ORR and CR rates reached 90% and 82%, respectively. With a median follow-up of 15 months, these responses were durable, with 1-year PFS and OS rates of 85% and 90%, respectively. The regimen also demonstrated a favourable tolerability profile, with low-grade CRS and infectious complications representing the most frequently reported and clinically relevant toxicities [76].

### 3.3. Tafasitamab Plus Lenalidomide and Rituximab (TLR)

A fully chemo-free scheme combining tafasitamab, lenalidomide, and rituximab (TLR) has been tested as a first-line therapy for older DLBCL patients (≥80 years old) in the phase 2 VERLen trial (NCT04974216). In the primary analysis including 67 older, highly comorbid patients with a median age of 84 years (range 80–91), TLR achieved an ORR of 71.6% (primary end point) including a CR rate of 49.3%. At a median follow-up of 16 months, the estimated 1-year PFS and 1-year OS were 52% and 82%, respectively. As expected in such a frail population, serious AEs occurred in more than half of the treated patients, predominantly neutropenia and infectious complications, while approximately one-third of patients permanently discontinued treatment, mainly because of disease progression. These data suggest that a completely chemo-free upfront strategy is feasible and may offer the potential for cure in older patients with DLBCL. Importantly, most patients experiencing relapse or progression later received salvage R-miniCHOP, achieving an ORR of 45%, suggesting that prior exposure to the TLR combination did not appear to abolish subsequent sensitivity to attenuated immunochemotherapy [77].

Of interest, a therapeutic approach combining rituximab and lenalidomide has already been investigated in the FIL_ReRi phase 2 study (NCT02955823; median age 83 years, range 70–91), showing favourable results and supporting further investigation of chemo-free strategies [78]. Given these findings, the ongoing phase 2 FIL_RICCO trial (NCT06835530) is evaluating rituximab plus golcadomide, a next-generation immunomodulatory agent, as frontline therapy for frail older patients with DLBCL.

### 3.4. Epcoritamab as Frontline Strategy

The multicenter phase 2 EPCORE DLBCL-3 trial (NCT05660967) recently investigated a fixed-duration regimen of 12 cycles of epcoritamab in older, highly comorbid patients considered ineligible for anthracycline-based immunochemotherapy. In the primary analysis, including 66 patients (median age 82.5 years, IQR 80–87) treated with epcoritamab monotherapy across stages 1 and 2, the ORR was 66.7%, with a CR rate of 57.6%. The median time to response was 1.5 months, whereas, with a median follow-up of 21.9 months the median DOR had not yet been reached. An early complete response emerged as a favourable prognostic factor. Estimated 1-year PFS and 1-year OS rates were 50.1% and 66.3%, respectively. Overall, the safety profile reflected the clinical frailty of the study population, with AEs reported in nearly all patients. The most common toxicities included CRS (71%), infections (68%), gastrointestinal disorders (42%), fatigue (23%), and peripheral edema (21%). ICANS occurred in 18% of patients, was more frequently observed among the oldest individuals, and, similarly to CRS, was predominantly low-grade, rapidly reversible, and temporally associated with the first full dose of epcoritamab.

The trial also evaluated epcoritamab plus lenalidomide in stage 1, in which 44 patients (median age 83 years, IQR 80.5–85) were assigned to receive the combination. The ORR and CR rates were numerically lower with epcoritamab plus lenalidomide than with monotherapy (59.1% and 45.5% vs. 70.5% and 63.6%, respectively). The combination was not selected for expansion into stage 2, based on the overall assessment of response rates and tolerability. Indeed, grade ≥ 3 treatment-emergent AEs occurred in 98% of patients receiving the combination, compared with 66% in the stage 1 monotherapy arm; serious AEs were reported in 81% and 64%, respectively. Grade ≥ 3 neutropenia and infections were also more frequent with the combination (40% and 38%, respectively) than with monotherapy (9% and 18%, respectively). Discontinuation due to AEs occurred in 40% of patients receiving epcoritamab plus lenalidomide versus 23% receiving monotherapy. Thus, these findings favoured further evaluation of monotherapy, although differences in baseline disease characteristics and the limited sample size preclude definitive conclusions [79].

Although reduced-intensity immunochemotherapy currently remains the standard first-line treatment for older patients with DLBCL, several ongoing clinical trials are investigating chemotherapy-sparing or completely chemotherapy-free strategies aimed at minimizing treatment-related toxicity while preserving efficacy. The most relevant ongoing trials are summarized in Table 1.

The key characteristics and safety outcomes of main clinical trials discussed within this review are summarized in Table 2.

## 4. Conclusions and Future Directions

The outcomes of older patients with DLBCL have improved substantially over the past few years following the approval of several novel therapies for the R/R setting.

Pola-BR and Tafa-Len represented the first major therapeutic advances and became the SOC for CAR-T cell-ineligible patients with R/R DLBCL from the second-line setting onward. The approval of CD20 × CD3 BsAbs in the third and later lines has further widened the therapeutic chances. More recently, Glofi-GemOx has moved BsAbs into the second-line setting in some countries. Lonca, targeting a different antigen, provides an additional alternative to BsAbs in the third line setting.

The use of anti-CD19 CAR-T therapy is expected to expand to older and frailer patients, supported by dedicated studies that already demonstrated a favourable safety profile and efficacy comparable to that observed in younger patients. In addition, next generation CAR-T products, such as the dual-target CD19/CD20 construct zamto-cel, might increase cellular therapy efficacy in these patients, while maintaining low toxicities.

Finally, several ambitious strategies currently under investigation, including TLR, Pola-R-Glo, and BsAbs monotherapy, may eventually represent a valid alternative to frontline standard immunochemotherapy for selected frail patients.

Although novel approaches may reduce the burden of conventional chemotherapy, they are not devoid of relevant toxicities; their use requires close monitoring and appropriate supportive care, including vaccination, antimicrobial prophylaxis, and immunoglobulin replacement according to current guidelines and individual clinical needs. Overall, the future of DLBCL treatment in older patients is likely to rely on individualized, fitness-adapted strategies rather than a universal low-intensity or chemotherapy-free paradigm. Further prospective studies with mature long-term survival outcomes, geriatric and quality-of-life endpoints, and evidence-based treatment-sequencing strategies are needed to define the optimal use of the expanding therapeutic armamentarium in this heterogeneous population.

Beyond treatment individualization, the optimal sequencing of available therapies represents a major challenge, particularly in R/R DLBCL. Treatment decisions should be guided by disease reassessment, ideally including re-biopsy to evaluate antigen expression, and careful consideration of prior therapies, as they can impact overall efficacy and immune competence. In particular, previous exposure to CD19-, CD20-, or CD79b-directed agents as well as to lymphotoxic chemotherapeutic agents such as bendamustine may influence the selection and timing of subsequent therapies. Treatment selection should also account for fixed-duration versus continuous treatment schedules, caregiver availability, expected adverse events—particularly infectious complications—and hospitalization requirements, whether mandated by regulatory provisions or by clinical needs. Nevertheless, the optimal sequencing of CAR-T cells, BsAbs, and ADCs, including their use as bridging strategies, remains an unresolved question requiring further evidence.

The current treatment algorithm for older patients with DLBCL, together with our perspective on future paradigm shifts, are summarized in Figure 1 and Figure 2, respectively, whereas the main therapeutic targets and strategies in DLBCL are illustrated in Figure 3.

## Figures and Tables

**Figure 1:**
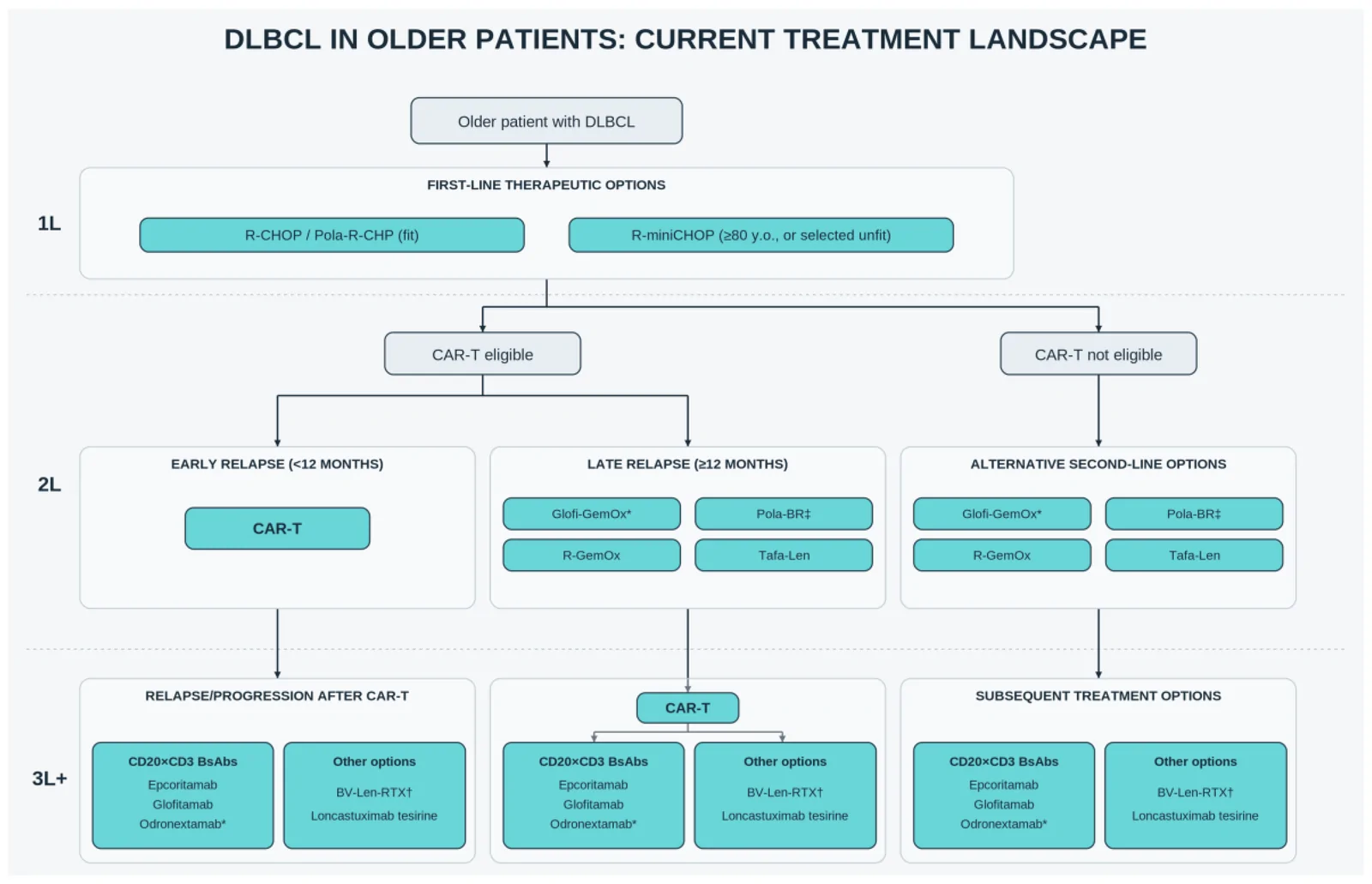
Current treatment landscape of diffuse large B-cell lymphoma in older patients. Illustrative overview of first-line and relapsed/refractory treatment options according to patient fitness, timing of relapse, and eligibility for CAR-T therapy. In the first-line setting, treatment intensity is adapted to age, comorbidities, and fitness. Subsequent treatment choices depend on prior exposure, patient characteristics, and local availability. The diagram represents a practical treatment framework rather than a formal guideline algorithm. Regulatory status: Symbols refer to approvals for the specified DLBCL indication and treatment setting: * approved by the EMA, but not by the FDA; † approved by the FDA, but not by the EMA; ‡ Pola-BR is approved from second-line therapy in the EU and after at least two prior treatment lines in the US. The FDA indication for BV-Len-RTX requires at least two prior systemic therapies and ineligibility for autologous stem cell transplantation or CAR-T therapy. Abbreviations: 1L, first-line therapy; 2L, second-line therapy; 3L+, third-line or later therapy; BsAbs, bispecific antibodies; BV-Len-RTX, brentuximab vedotin plus lenalidomide and rituximab; CAR-T, chimeric antigen receptor T-cell therapy; DLBCL, diffuse large B-cell lymphoma; EMA, European Medicines Agency; FDA, U.S. Food and Drug Administration; Glofi-GemOx, glofitamab plus gemcitabine and oxaliplatin; Pola-BR, polatuzumab vedotin plus bendamustine and rituximab; Pola-R-CHP, polatuzumab vedotin, rituximab, cyclophosphamide, doxorubicin, and prednisone; R-CHOP, rituximab, cyclophosphamide, doxorubicin, vincristine, and prednisone; R-GemOx, rituximab plus gemcitabine and oxaliplatin; R-miniCHOP, reduced-intensity R-CHOP; Tafa-Len, tafasitamab plus lenalidomide; y.o., years old. Figure created using R software (version 4.6.1; R Foundation for Statistical Computing, Vienna, Austria).

**Figure 2:**
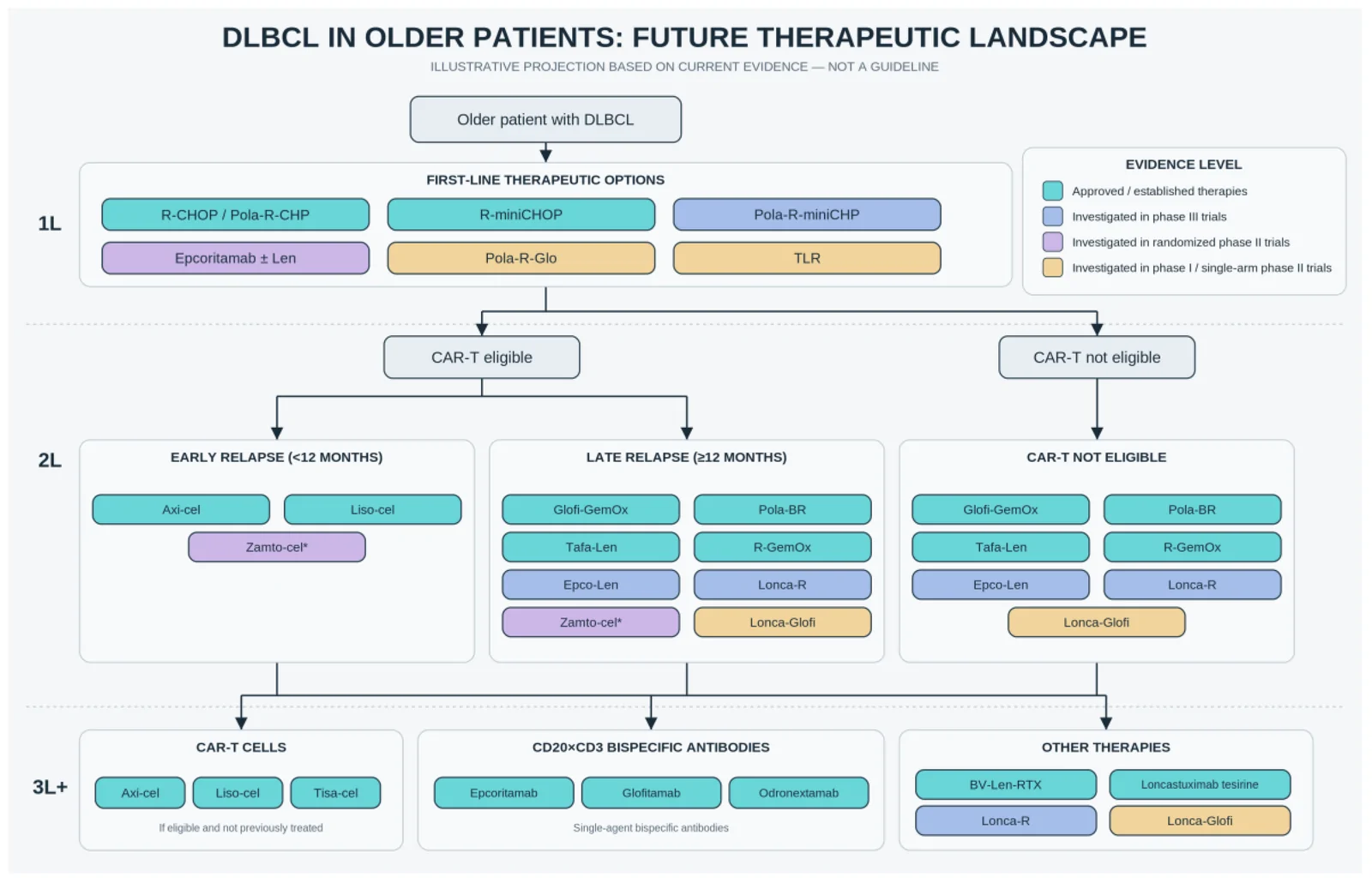
Emerging and future therapeutic landscape of diffuse large B-cell lymphoma in older patients. Illustrative projection of established and investigational therapeutic strategies for older patients with DLBCL, organized by treatment line, fitness, CAR-T eligibility, and timing of relapse. Colors distinguish approved/established options (turquoise) from strategies investigated in phase III (blue), randomized phase II (purple), or phase I/single-arm phase II trials (yellow). These categories reflect the phase of clinical investigation in the specified setting, not necessarily the availability of mature efficacy results. The figure is not a clinical practice guideline or a validated treatment-sequencing algorithm. * Zamto-cel was investigated in the randomized DALY 2-EU trial in transplant-ineligible patients with refractory disease or relapse within 24 months from initiation of first-line therapy. Abbreviations: 1L, first-line therapy; 2L, second-line therapy; 3L+, third-line or later therapy; Axi-cel, axicabtagene ciloleucel; BsAbs, bispecific antibodies; BV-Len-RTX, brentuximab vedotin plus lenalidomide and rituximab; CAR-T, chimeric antigen receptor T-cell therapy; DLBCL, diffuse large B-cell lymphoma; Epco-Len, epcoritamab plus lenalidomide; Glofi-GemOx, glofitamab plus gemcitabine and oxaliplatin; Len, lenalidomide; Liso-cel, lisocabtagene maraleucel; Lonca-Glofi, loncastuximab tesirine plus glofitamab; Lonca-R, loncastuximab tesirine plus rituximab; Pola-BR, polatuzumab vedotin plus bendamustine and rituximab; Pola-R-CHP, polatuzumab vedotin, rituximab, cyclophosphamide, doxorubicin, and prednisone; Pola-R-Glo, polatuzumab vedotin plus rituximab and glofitamab; Pola-R-miniCHP, polatuzumab vedotin plus reduced-intensity rituximab, cyclophosphamide, doxorubicin, and prednisone; R-CHOP, rituximab, cyclophosphamide, doxorubicin, vincristine, and prednisone; R-GemOx, rituximab plus gemcitabine and oxaliplatin; R-miniCHOP, reduced-intensity R-CHOP; Tafa-Len, tafasitamab plus lenalidomide; Tisa-cel, tisagenlecleucel; TLR, tafasitamab plus lenalidomide and rituximab; Zamto-cel, zamtocabtagene autoleucel. Figure created using R software (version 4.6.1; R Foundation for Statistical Computing, Vienna, Austria).

**Figure 3:**
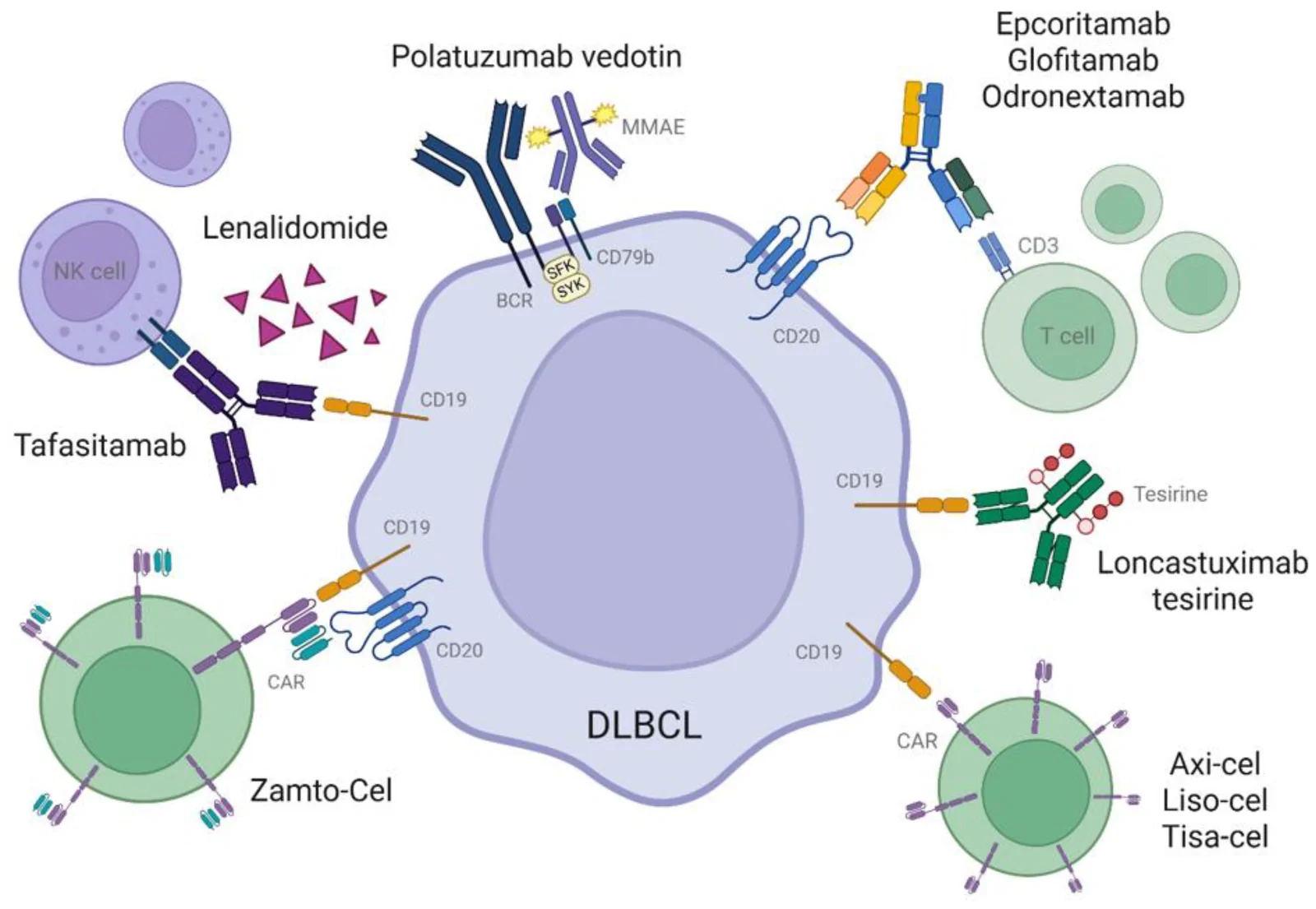
Therapeutic targets and targeted strategies in diffuse large B-cell lymphoma. Graphical representation of a DLBCL cell, its key surface receptors, and emerging therapeutic strategies targeting these molecules. Abbreviations: CAR, chimeric antigen receptor; BCR, B-cell receptor; CD, cluster of differentiation; DLBCL, diffuse large B-cell lymphoma; MMAE, monomethyl auristatin E; NK, natural killer; SFK, Src family kinase; SYK, spleen tyrosine kinase; Zamto-cel, zamtocabtagene autoleucel. Figure created with BioRender.

**Table 1:** Selected clinical trials evaluating novel frontline therapeutic strategies in older and/or frail patients with diffuse large B-cell lymphoma.

NCT Number	Intervention	Phase	Status	Study Population	Primary Endpoint
NCT06176729	Polatuzumab vedotin + rituximab + lenalidomide	2	Recruiting	Age ≥ 70; unfit/frail by CGA; previously untreated DLBCL	CR rate
NCT04974216	Tafasitamab + rituximab + lenalidomide (VERLen)	2	Active, not recruiting	Age ≥ 80; previously untreated DLBCL	ORR
NCT07012980	Glofitamab + zanubrutinib + polatuzumab vedotin	2	Recruiting	Age ≥ 70; previously untreated DLBCL	PFS
NCT05798156	Glofitamab + polatuzumab vedotin + rituximab (Pola-R-Glo)	2	Active, not recruiting	Age > 60; ineligible for full-dose R-CHOP; previously untreated aggressive B-cell lymphoma	PFS
NCT05207670	Mosunetuzumab (MorningSun, DLBCL cohort)	2	Active, not recruiting	Age ≥ 80, or age 65–79 and ineligible for full-dose R-CHOP due to functional impairment (ADL/IADL) or comorbidities (CIRS-G); previously untreated DLBCL	PFS
NCT06758037	Zebtorizumab + lenalidomide + NGS-guided target therapy	2	Recruiting	Age ≥ 70; unfit/frail by sGA; previously untreated DLBCL	CR rate
NCT06835530	Golcadomide + rituximab (FIL_RICCO)	2	Recruiting	Age ≥ 80; frail by sGA; previously untreated DLBCL	PFS
NCT07072208	Pomalidomide + orelabrutinib + rituximab → response-adapted addition of polatuzumab vedotin	NA	Recruiting	Age ≥ 70; previously untreated DLBCL	CR rate
NCT07397832	Chidamide + polatuzumab vedotin + rituximab (CRP)	2	Recruiting	Age ≥ 70, or age 60–69 with ECOG PS 2–4; previously untreated double-expressor DLBCL	CR rate
NCT07415980	Chidamide + polatuzumab vedotin + rituximab	1b/2	Not yet recruiting	Age ≥ 70, or age 60–69 with frailty by CGA; previously untreated double-expressor DLBCL	DLT and recommended phase 2 dose (phase 1b); CR rate (phase 2)
NCT06764017	Chidamide + zanubrutinib + lenalidomide (CHiR)	2	Recruiting	Age ≥ 70; unfit/frail by sGA and ineligible for full-dose R-CHOP; previously untreated double-expressor DLBCL	CR rate
NCT05952024	Acalabrutinib + rituximab (ACRUE)	2	Recruiting	Age ≥ 80, or age 65–79 and ineligible for standard immunochemotherapy; previously untreated DLBCL	Grade 3–4 TEAEs rate
NCT06530511	Orelabrutinib + polatuzumab vedotin + rituximab (PRO)	2	Unknown †	Age ≥ 60; unfit/frail by sGA; previously untreated non-GCB DLBCL	CR rate
NCT06683885	Orelabrutinib OR decitabine + rituximab + oral cyclophosphamide + prednisone	1/2	Recruiting	Age ≥ 65; ineligible for standard immunochemotherapy; previously untreated DLBCL	ORR and PFS (co-primary endpoints)
NCT06522555	Zanubrutinib + polatuzumab vedotin + rituximab + lenalidomide (Pola-ZR2) vs. ZR2 (POTENT)	3	Recruiting	Age ≥ 80, or 70–79 with frailty by CGA; previously untreated DLBCL	PFS
NCT04460248	Zanubrutinib + rituximab + lenalidomide (ZR2)	2	Active, not recruiting	Age ≥ 75, or unfit/frail by CGA; previously untreated DLBCL	CR rate
NCT06778902	Azacitidine + R-GemOx	2	Not yet recruiting	Age ≥ 60; previously untreated DLBCL	ORR
NCT02734771	Brentuximab vedotin + R-miniCHP	2	Active, not recruiting	Age ≥ 75; previously untreated DLBCL	Proportion completing six cycles of therapy
NCT04332822	Polatuzumab vedotin + R-miniCHP vs. R-miniCHOP (POLAR BEAR)	3	Recruiting	Age ≥ 80, or ≥75 frail by sGA; previously untreated DLBCL	PFS
NCT04594798	Polatuzumab vedotin + R-miniCHP	2	Active, not recruiting	Age ≥ 75; previously untreated DLBCL	PFS
NCT06828991	Polatuzumab vedotin + R-miniCHP → ctDNA-guided mosunetuzumab consolidation	2	Recruiting	Age ≥ 70, ineligible for full dose immunochemotherapy; previously untreated DLBCL	ctDNA clearance rate after mosunetuzumab consolidation
NCT07714421	Epcoritamab induction → epcoritamab + Pola-R-miniCHP	1	Not yet recruiting	Age > 80, or 70–79 ineligible for full-dose R-CHOP due to functional impairment (ADL/IADL) or comorbidities (CIRS-G); previously untreated DLBCL	PFS
NCT06765317	Glofitamab + polatuzumab vedotin + R-miniCHP (GLORY)	2	Recruiting	Age ≥ 80, or 65–79 unfit/frail by sGA; previously untreated DLBCL	CR rate
NCT06594939	Mosunetuzumab + polatuzumab vedotin + split-dose CHP	2	Recruiting	Age ≥ 75, or 70–74 unfit/frail by CIRS-G; previously untreated DLBCL	CR rate
NCT07552324	Chidamide + lisaftoclax + polatuzumab vedotin + R-CHP	2	Not yet recruiting	Age ≥ 65; previously untreated double-hit/triple-hit DLBCL	CR rate
NCT06674096	Rituximab + lenalidomide + chidamide → CR-CHOP → chidamide maintenance	2	Recruiting	Age > 70, or age ≥ 65 with ECOG PS ≥ 2; previously untreated double-expressor DLBCL	PFS
NCT07493148	Chidamide + R-miniCHOP → chidamide + rituximab maintenance	2	Recruiting	Age ≥ 70; previously untreated double-expressor DLBCL	PFS
NCT05820841	Acalabrutinib + R-miniCHOP vs. R-miniCHOP (ARCHED)	3	Recruiting	Age > 80, or age > 60 to ≤80 and ineligible for full-dose R-CHOP; previously untreated DLBCL	PFS
NCT04799275	Azacitidine + R-miniCHOP vs. R-miniCHOP (SWOG S1918)	2/3	Active, not recruiting	Age ≥ 75; previously untreated DLBCL	PFS (phase 2); OS (phase 3)

Abbreviations: ADL, activities of daily living; CGA, comprehensive geriatric assessment; CHiR, chidamide, zanubrutinib, and lenalidomide; CHP, cyclophosphamide, doxorubicin, and prednisone; CIRS-G, Cumulative Illness Rating Scale–Geriatric; CR, complete response; CR-CHOP, chidamide, rituximab, cyclophosphamide, epirubicin (or liposomal doxorubicin), vindesine, and prednisone (as defined in NCT06674096); CRP, chidamide, rituximab, and polatuzumab vedotin; ctDNA, circulating tumor DNA; DLBCL, diffuse large B-cell lymphoma; DLT, dose-limiting toxicity; ECOG PS, Eastern Cooperative Oncology Group performance status; GCB, germinal center B-cell; IADL, instrumental activities of daily living; NA, not available; NCT, ClinicalTrials.gov identifier; NGS, next-generation sequencing; ORR, overall response rate; OS, overall survival; PFS, progression-free survival; R-CHOP, rituximab, cyclophosphamide, doxorubicin, vincristine, and prednisone; R-CHP, rituximab, cyclophosphamide, doxorubicin, and prednisone; R-GemOx, rituximab, gemcitabine, and oxaliplatin; R-miniCHOP, reduced-intensity R-CHOP; R-miniCHP, reduced-intensity rituximab, cyclophosphamide, doxorubicin, and prednisone; sGA, simplified geriatric assessment; TEAEs, treatment-emergent adverse events; ZR2, zanubrutinib, rituximab, and lenalidomide. † Unknown indicates a study whose last known status was recruiting, not yet recruiting, or active, not recruiting, which has passed its completion date and whose status has not been verified within the preceding two years, according to ClinicalTrials.gov.

**Table 2:** The key characteristics and safety outcomes of the experimental arm within selected clinical trials discussed in this review.

Reference	Experimental Intervention	Age Median (Range)	≥70 Years	≥75 Years	Frailty Criteria or Geriatric Assessment	AEs of Special Interest G3–4	Infections G3–4	Cytopenias G3–4	TRM	Discontinuation Due to AEs
Coiffier B *NEJM* (2002)	R-CHOP ^1^	69 (60–80)	50%	24%	-	PN: 5% Cardiotox: 8%	12%	A: 14% T: NR N: NR	10.4%	NR
Peyrade F *Lancet Onc* (2011)	R-miniCHOP ^1^	83 (80–95)	100%	100%	IADL evaluation	PN: 7% Cardiotox: 4%	13%	A: 9% T: 7% N: 40%	16.8%	7.4%
Tilly H * NEJM (2022)	Pola-R-CHP ^1^	65 (19–80)	32% ^†^	13.6% ^†^	-	PN: 1.6% Cardiotox: NR	15.2%	A: 12% T: NR N: 28.3%	3%	6.2%
Sekiguchi Y * JCEH (2023)	Pola-R- mini-CHP ^1^	83 (80–92)	100%	100%	-	PN: 0% Cardiotox: NR	8.7%	A: 17.4% T: 4.3% N: 17.4%	4.3%	13%
Sehn LH *JCO* (2020)	Pola-BR ^1^	67 (33–86)	NR (≥65 years: 58%)	NR	-	PN: 0%	23.1%	A: 28.2% T: 41% N: 46.2%	12.8%	33.3%
Salles G *Lancet Onc* (2020)	Tafa-Len ^2^	72 (41–86)	56%	NR	-	Diarrhea: 1.2% Cutaneous: 8.6%	~25%	A: 7.4% T: 17.3% N: 48.1%	14.8% ^†^	19.8% Tafa ^†^ 22.2% Len ^†^
Caimi PF * Haematologica (2024)	Lonca ^2^	66 (23–94)	NR (≥65 years: 55%)	14.5%	-	Hepatotox: 20.7% Cutaneous: 3.5%	NR	A: 10.3% T:17.9% N: 26.2%	NR	24.8%
Carlo-Stella C * CLML (2025)	Lonca-R ^1^	A: 84 (80–91) B: 80 (67–92)	NR	A: 100%	sGA (age, ADL, IADL) +CIRS-G unfit(A)-frail(B)	Any G ≥3 AE: 75.6%	NR	NR	26.8%	NR
Gini G *Blood* (2023)	R-Len ^1^	83 (70–91)	100%	NR	sGA (ADL, IADL, CIRS-G); all frail	Diarrhea: 4.6% Cutaneous: 11%	10%	A: 3.1% T: 9.2% N: 46.1%	27.7%	21.5%
Bartlett NL * JCO (2025)	BV-Len-R ^2^	74 (29–87)	NR	NR	-	PN: 6% Diarrhea: 4% Cutaneous: NR	NR	A: 22% T: 25% N: 43%	12%	15%
Houot R * NRtMed (2023)	Axi-cel (ALYCANTE) ^3^	70 (49–81)	53.2%	11.3%	-	CRS: 8.1% ICANS: 14.5%	17.7%	A: 38.7% T: 38.7% N: 66.1%	9.7%	-
Sehgal A * Blood Adv (2025)	Liso-cel (PILOT) ^3^	74 (53–84)	79%	46%	-	CRS: 2% ICANS: 5%	7%	A: 11% T: 21% N: 48%	3.3%	-
Schuster SJ * NEJM (2019)	Tisa-cel ^3^	56 (22–76)	NR (≥65 years: 23%)	NR	-	CRS: 22% ICANS: 12%	20%	A: 39% T: 28% N: 33%	NR	-
Borchmann P * Blood (2025)	Zamto-cel ^3^	74 (19–87)	NR	NR	-	CRS: 5.3% ICANS: 1.3%	NR	NR	NR	-
Dickinson MJ * NEJM (2022)	Glofi ^1^	66 (21–90)	NR	NR	-	CRS: 4% ICANS: 3%	15%	A: 6% T: 8% N: 27%	5%	9%
Thieblemont C * JCO (2023)	Epco ^2^	64 (20–83)	NR (≥65 years: 49.1%)	18.5%	-	CRS: 2.5% ICANS: 0%	26% ^†^	A: 10.2% T: 5.7% N: 14.6%	NR	7.6%
Kim WS * NRt Cancer (2025)	Odro ^2^	67 (24–88)	NR	23.6%	-	CRS: 4.7% ICANS: 0%	26.8%	A: 22.8% T: 15% N: 26%	15.7%	13.4%
Abramson JS *The Lancet* (2024)	Glofi-GemOx ^1^	68 (22–88)	NR (≥65 years: 63.4%)	25.1%	-	CRS: 2.3% ICANS: 0.6% PN: NR	16.9%	A: NR T: NR N: NR	8.9% ^†^	23.3% ^†^
Gurion R * Blood (2024)	Epco-Len ^1^	71.5 (26–85)	NR	NR	-	CRS: 10% ICANS: 2.5% Diarrhea: NR Cutaneous: NR	NR	A: 20% T: 17.5% N: 60%	2.5%	10%
Alderuccio JP * EHA (2025)	Lonca-Glofi ^1^	71 (26–85)	NR	NR	-	CRS: 2.4% ICANS: 0% Hepatotox: 14.6% Cutaneous: 2.4%	7.3%	A: 9.8% T: 7.3% N: 24.4%	NR	7.3%
Chapuy B *Blood* (2025)	R-Pola-Glofi ^1^	80 (NR)	NR	NR	-	NR	NR	NR	NR	NR
Vitolo U * Lancet Haem (2026)	Epco-Len ^1^	83	100%	100%	Age ≥80 years, or ≥75 years with relevant comorbidity	NR	38%	A: NR T: NR N: 40%	14%	NR
Budde LE * JCO (2025)	Mosu-Pola ^1^	62(23–87)	NR	NR	-	CRS: 0.7% ICANS: 0% PN: 0%	16%	A: 5.9% T: 2.2% N: 33%	5.2%	2.2%

Abbreviations: A, Anemia; ADL, Activities of Daily Living; AEs, Adverse Events; Axi-cel, Axicabtagene ciloleucel; BR, Bendamustine + Rituximab; BV, Brentuximab Vedotin; Cardiotox, Cardiotoxicity; CHOP, Cyclophosphamide + vincristine + prednisone + doxorubicin; CHP, Cyclophosphamide + prednisone + doxorubicin; CIRS-G, Cumulative Illness Rating Scale-Geriatric; CRS, Cytokine Release Syndrome; Epco, Epcoritamab; GemOx, Gemcitabine + Oxaliplatin; Glofi, Glofitamab; G, Grade; Hepatotox, Hepatotoxicity; HGBCL, High Grade B-cell Lymphoma; IADL, Instrumental activities of Daily living; ICANS, Immune Effector Cell-Associated Neurotoxicity Syndrome; Len, LeNRlidomide; Liso-cel, Lisocabtagene maraleucel; Lonca, Loncastuximab tesirine; Mosu, Mosunetuzumab; N, Neutropenia; NR, Not Reported; Odro, Odronextamab: PN, Peripheral Neuropathy; Pola, Polatuzumab Vedotin; R, Rituximab; sGA, Simplified Geriatric Assessment; T, Thrombocytopenia; Tafa, Tafasitamab; Tisa-cel, Tisagenlecleucel; TRM; Treatment Related Mortality; Zamto-cel, Zamtocabtagene autoleucel. ^†^ Value derived from another publication of the same trial. * HGBCL includable. ^1^ Fixed duration treatment. ^2^ Treatment until progression or unacceptable toxicity. ^3^ Single infusion.

## Data Availability

No new data were created or analyzed in this study. Data sharing is not applicable to this article.
